# Application of implementation science framework to develop and adopt regulatory science in different national regulatory authorities

**DOI:** 10.3389/fpubh.2023.1172557

**Published:** 2023-05-04

**Authors:** Junnan Shi, Xianwen Chen, Hao Hu, Carolina Oi Lam Ung

**Affiliations:** ^1^State Key Laboratory of Quality Research in Chinese Medicine, Institute of Chinese Medical Sciences, University of Macau, Taipa, Macao SAR, China; ^2^Centre for Pharmaceutical Regulatory Sciences, University of Macau, Taipa, Macao SAR, China; ^3^Department of Public Health and Medicinal Administration, Faculty of Health Sciences, University of Macau, Taipa, Macao SAR, China

**Keywords:** PRECEDE-PROCEED model (PPM), regulatory science, implementation science, drug regulatory authorities (DRAs), The United States (US), European Union, Japan, China

## Abstract

**Introduction:**

The purpose of developing and adopting regulatory science (RS) for drug regulatory authorities (DRAs) is to enhance regulatory capacity by advancing the scientific approach for the evaluation of health-related products. While many DRAs around the world advocate the concept of RS, the implementation approaches of RS vary according to local needs and have not been systemically examined. This study aimed to systematically identify the evidence about how RS was developed, adopted, and advanced by the selected DRAs, and analyzed and compared the implementation experiences of RS development under the guidance of an implementation science framework.

**Methods:**

Documentary analysis of government documents and a scoping literature review were conducted, and data analysis was performed under the guidance of the PRECEDE-PROCEED Model (PPM). DRAs in the United States, the European Union, Japan, and China had officially launched RS initiatives and were therefore selected as the target countries in this study.

**Results:**

There is no common consensus on the definition of RS among the DRAs. However, these DRAs shared the same goal of developing and adopting RS, which was used to develop new tools, standards, and guidelines that could improve the effectiveness and efficiency of the risk and benefit assessment of the regulated products. Each DRA had decided its own priority areas for RS development and thus set specific objectives that might be technology-based (e.g., toxicology and clinical evaluation), process-based (e.g., partnership with healthcare systems and high-quality review/consultation services), or product-based (e.g., drug-device combination products and innovative emerging technologies). To advance RS, considerable resources had been allocated for staff training, advancing information technology and laboratory infrastructure, and funding research projects. DRAs also took multifaceted approaches to expand scientific collaborations through public–private partnerships, research funding mechanisms, and innovation networks. Cross-DRA communications were also reinforced through horizon scanning systems and consortiums to better inform and assist the regulatory decision-making process. The output measurements might be scientific publications, funded projects, DRAs interactions, and evaluation methods and guidelines. Improved regulatory efficiency and transparency leading to benefits to public health, patient outcomes, and translation of drug research and development as the key primary outcomes of RS development were anticipated but not yet clearly defined.

**Conclusion:**

The application of the implementation science framework is useful for conceptualizing and planning the development and adoption of RS for evidence-based regulatory decision-making. Continuous commitment to the RS development and regular review of the RS goals by the decision-makers are important for DRAs to meet the ever-changing scientific challenges in their regulatory decision-making process.

## 1. Introduction

The acceleration of innovation is catalyzing the development of increasingly complex pharmaceutical products and emerging technologies, such as cell and gene therapies, drug–device combinations, artificial intelligence, and digital health. These come with vast opportunities to promote, maintain, and protect human health but also some significant challenging regulatory issues to national drug regulatory authorities (DRAs) (1, 2). Beyond science and technological innovation, as shown by the COVID-19 pandemic, drug regulation has seen challenges in engaging collaborative effort and formulating regulatory flexibility in advancing responses to unexpected public health threats (3). Regulators play an integral role in the complex ecosystem of the pharmaceutical system and often face the challenges of meeting societal expectations to provide patients with timely access to new treatment options while maintaining stakeholder trust by evidently upholding high regulatory practice standards (4).

Regulatory science (RS) is a scientific discipline that embraces a large variety of activities and outputs, from new techniques and products to methodological standards and guidance (5). In order to address the above challenges, major national DRAs have proposed and adopted this cutting-edge discipline. Successful outcomes had been demonstrated by the development of new tools, standards, and approaches (3, 6, 7).

**Figure 1:**
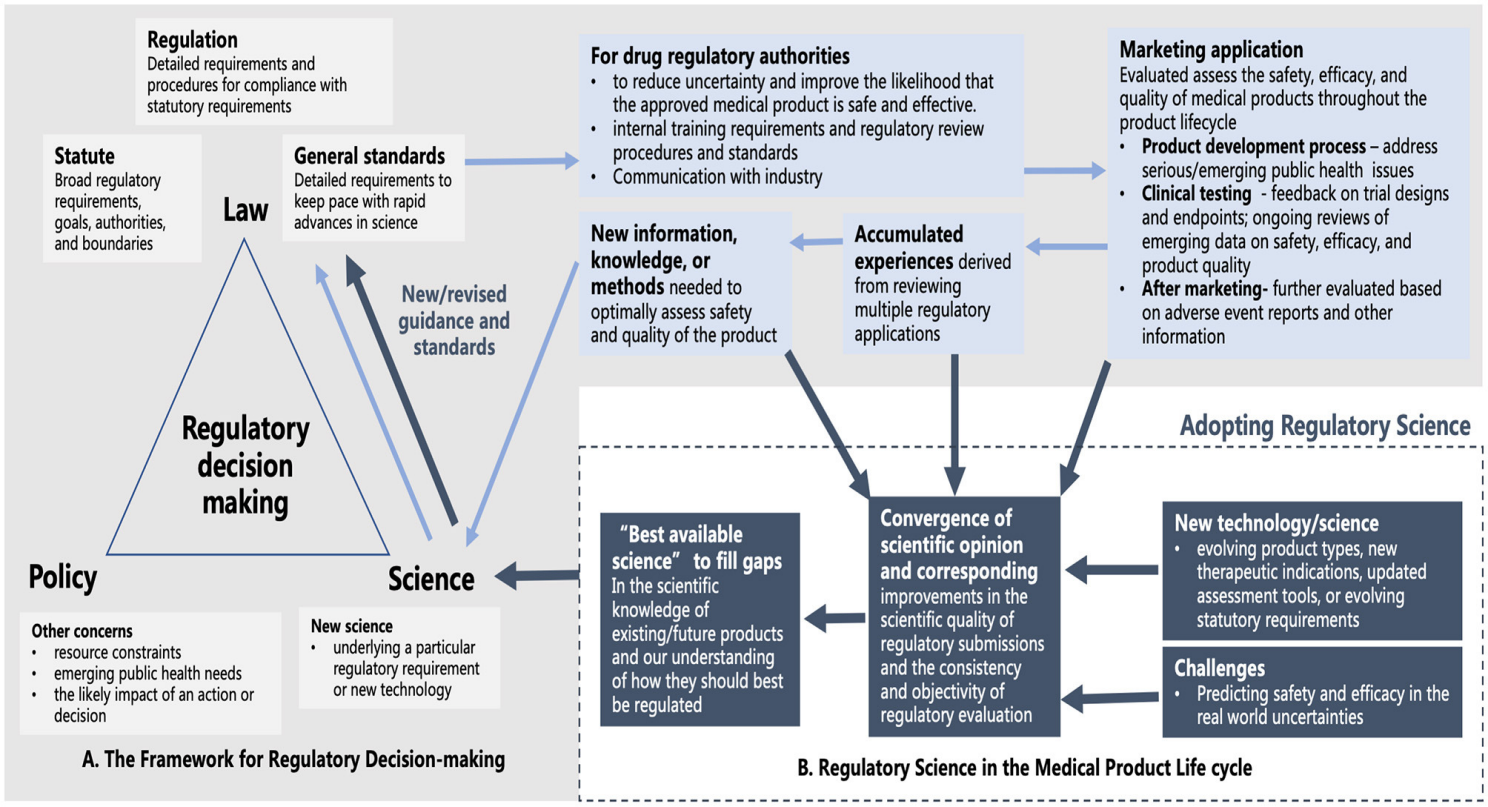
**(A)** The backbone of the FDA's regulatory decision-making process and the development of new information and knowledge based on the oversight of the medical product life cycle. **(B)** An additional source of scientific input for improving the quality, consistency, and objectivity of regulatory evaluation with the adoption of RS (Pale blue arrow: how new scientific knowledge is generated based on regulatory practice to support the regulatory decision-making process; Navy blue arrow: additional generation and uptake of scientific input to better inform regulatory decision-making upon adoption of RS).

The development of RS helps to bridge “law and regulation” and “science” more effectively in the interests of addressing healthcare needs and promoting innovation (8). For example, the backbone of the FDA's regulatory decision-making process is formed with policy, law, and science supplemented with guidance or standards to facilitate law implementation. By having regulatory measures overseeing the whole product life cycle, regulatory experiences and challenges accumulated lead to the development of new information and knowledge, and continuous improvement and revision of laws and regulations driven by advances in science and innovation (Figure 1A). By adopting RS, it helps the FDA make their regulatory practices in convergence with scientific opinions and identify the “best available science” to achieve improvements in the quality, consistency, and objectivity of regulatory evaluation more timely and effectively (Figure 1B).

Current evidence from literature about the development of RS mainly focused on the regulation of specific pharmaceutical product categories. For example, the International Neonatal Consortium (INC) addressed the need for measurement and assessment of clinical outcomes in neonates using cell-based therapies through public–private partnerships that shared data and expertise to advance RS (9). The Global Coalition for Regulatory Science Research (GCRSR) provided an overview of new tools and methodologies for regulatory bodies from various countries around the globe in nanotechnology and more particularly nanotechnology-based products (10). In addition, a research project on the harmonization and evaluation of regulations for pharmaceuticals and medical devices by the Japan Agency for Medical Research and Development has taken regulatory consideration of the shared data and information required for interoperable medical devices (11). Japanese scholars summarized the development experience of the ethical Kampo products with new dosage forms and herbal medicines that used Kampo extracts as active pharmaceutical ingredients, illustrating the importance of RS for the development of new drugs for natural products (12).

While most of the current literature explains how RS can lead to technological innovation or quality improvement, little research had been attended to the development and adoption process. In order to provide a tool for decision-makers for developing and adopting RS to support regulatory decision-making, the main objective of this study aimed to systematically identify the evidence about how RS was developed, adopted, and advanced by the selected DRAs, and analyzed and compared the implementation experiences of RS development under the guidance of the commonly accepted PRECEDE-PROCEED model derived from implementation science. It is anticipated that the results of this study will help inform DRAs and academia about how to promote the systematic and innovative application of RS under the guidance of a theoretical framework.

## 2. Materials and methods

This study adopted a critical review approach to obtain publicly available information about developing and adopting RS by different DRAs from government and official websites, as well as related literature from electronic databases. The collected data were extracted, compiled, and comparatively analyzed under the guidance of the PRECEDE-PROCEED model.

### 2.1. Theoretical framework

To obtain more evidence from theory to practical application, implementation science has been commonly applied to guide the process of developing and adopting RS to facilitate scientific regulatory decision-making (13). The theories, frameworks, and research of implementation science can be used to lead RS to the more specific focused field of pharmaceutical regulation (14). In particular, the PRECEDE-PROCEED model (PPM) has been fully demonstrated in many practices as a theoretical framework for the planning and evaluation of public health programs (15). It provides a visual display of a program to help guide the regulator's system thinking of the program development. PPM provides continuous efforts for the pre-intervention of goals and post-intervention monitoring and quality improvement. It allows the regulators to logically think about the desired endpoints and work “backwards” to achieve the goal (16). The outcome-oriented character promotes better policy decision-making to improve public health (17, 18).

**Figure 2:**
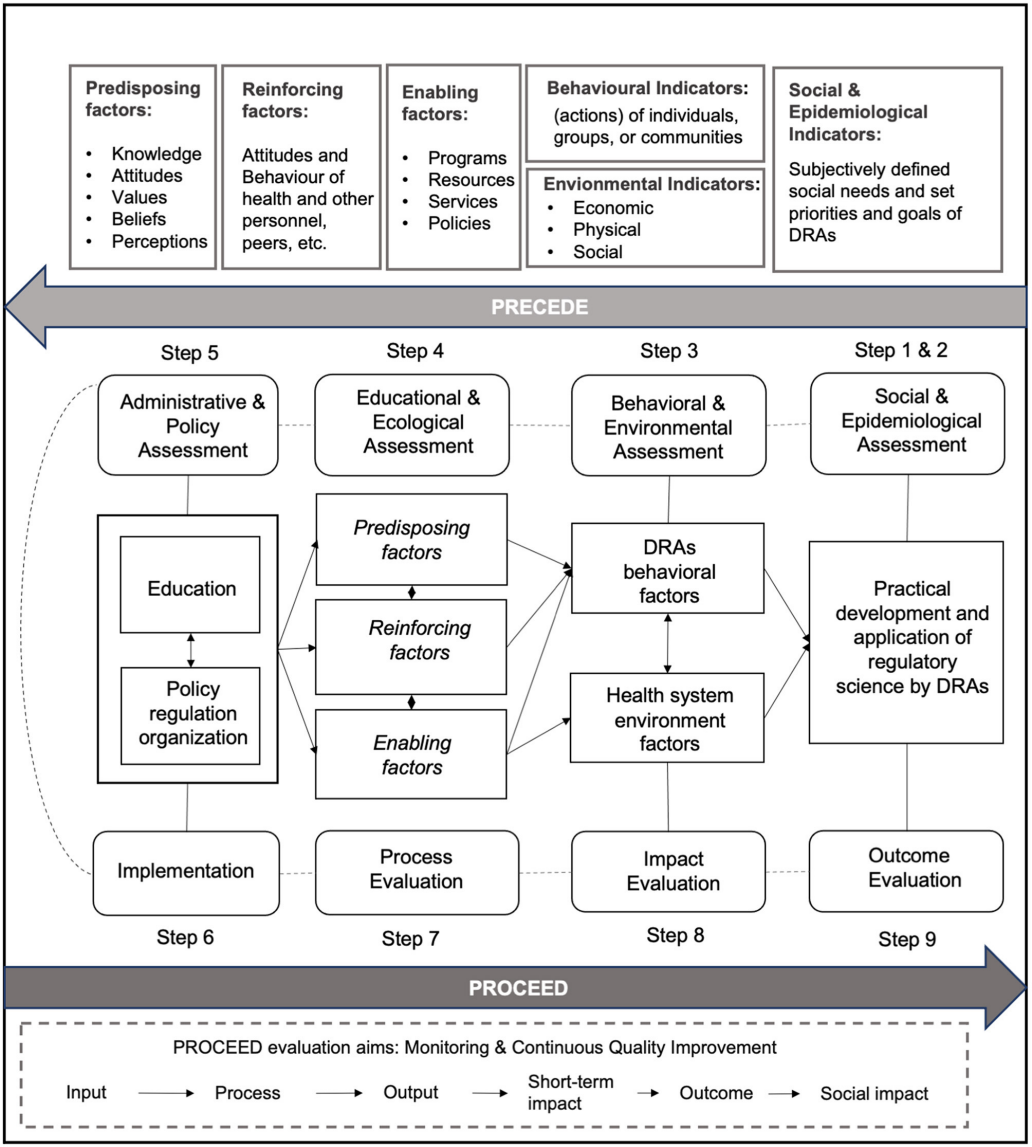
Overview of the PRECEDE-PROCEED model (PPM) applied to the implementation of regulatory science by DRAs.

As shown in Figure 2, in the PPM model, the PRECEDE phase consists of five steps. Steps 1 and 2: to determine the social needs of RS and then set priorities and goals of DRAs; Step 3: to determine the behavioral indicators (e.g., individuals or groups) of DRAs and environmental indicators (e.g., economic or physical) of the whole health system that have an impact on RS; Step 4: to determine the predisposing (e.g., knowledge or values), reinforcing (e.g., attitudes of health peers), and enabling factors (e.g., programs or policies) involved in the educational or ecological that have an impact on RS; Step 5: to determine the administrative and policy components of RS. The PROCEED phase consists of four steps to achieve the evaluation aim of monitoring and continuous quality improvement. Step 6: to evaluate the actual implementation (input) of developing and adopting RS; Step 7: to evaluate the process of advancing RS; Step 8: to evaluate the impact of advancing RS (output or short-term impact, if applicable); Step 9: to evaluate the outcomes of developing and adopting RS (social impact or long-term impact, if applicable).

### 2.2. Data retrieval and collection

The United States, the European Union, Japan, and China were targeted for evaluation because they all have officially released regulatory science programs. The data collection was conducted in December 2022 to search the publicly available documents and other related literature. Publicly available documents and reports were found from the government and official websites of the corresponding DRAs: National Medical Products Administration (NMPA) in China https://www.nmpa.gov.cn/, Food and Drug Administration (FDA) in the U.S. https://www.fda.gov/, European Medicines Agency (EMA) in the European Union https://www.ema.europa.eu/en, and the Pharmaceuticals and Medical Devices Agency in Japan (PMDA) http://www.pmda.go.jp/. Additionally, academic and gray literature was conducted using Google Scholar and PubMed. Each search used the following terms: [(regulatory science) OR (translational science) OR (regulation) OR (regulatory agenc^*^) OR (regulatory authorit^*^)] AND [(United States) OR (European Union) OR (Japan) OR (China) OR (FDA) OR (EMA) OR (PMDA) OR (NMPA)]. The search was conducted separately by two of the authors (JS and XC), and the documents and materials to be included for further analysis were cross-checked and confirmed by two other authors (HH and COLU).

### 2.3. Data extraction and analysis

The PPM provides structures to guide the extraction of data related to specific objectives and influencing factors before the implementation of intervention as well as a continuous quality improvement after the intervention is implemented. For eligible data, two authors (JS and XC) extracted and evaluated information independently and any disagreements were resolved by discussion or reaching an agreement with the third author (COLU). The two phases of PRECEDE and PROCEED in the PPPM were combined to form the complete theoretical data extraction. The authors (JS, COLU, and HH) adjusted construct definitions applicable to develop and adopt RS by adapting from PRECEDE-PROCEED steps defined by the framework. After adjudicating these applications for RS, the authors' team then refined the definitions accordingly. In addition, the construct definitions were adjusted for adoption in the context of RS in consultation with experienced researchers in drug regulation.

## 3. Results

As an overview, based on the PRECEDE section of the PPM, we sorted out the information on developing and adopting RS from PRECEDE to PROCEED of DRAs in selected countries. To advance RS, considerable resources allocated for staff training, advancing information technology and laboratory infrastructure, and funding research projects were also identified. DRAs also took multifaceted approaches to expand scientific collaborations through public–private partnerships, research funding mechanisms, and innovation networks. Cross-DRAs communications were also reinforced through a horizon scanning system and consortium to better inform and assist the regulatory decision-making process.

### 3.1. Step 1 and Step 2: social and Epidemiological diagnosis

As an overview, based on the PRECEDE section of the PPM, we sorted out the information on developing and adopting RS from PRECEDE to PROCEED of DRAs in selected countries. To advance RS, considerable resources had been allocated for staff training, advancing information technology and laboratory infrastructure, and funding research projects. DRAs also took multifaceted approaches to expand scientific collaborations through public–private partnerships, research funding mechanisms, and innovation networks. Cross-DRAs communications were also reinforced through a horizon scanning system and consortium to better inform and assist the regulatory decision-making process. For DRAs who opted to use a theoretical framework to effectively apply RS to scientific decision-making of the full life cycle of pharmaceutical products, identifying and assessing the various societal needs and priorities that influenced regulators to apply RS became an important first step toward achieving this goal.

#### 3.1.1. Social needs

Entering the 21st century, DRAs faced a series of public health challenges, including new industrial transformation and globalization brought about by technological innovation, new changes, and challenges caused by the rapid development of knowledge and research in the field of basic science. More specifically, gaps between advanced therapy medicinal products (ATMP) and the actual application of treatments to patients remained unsolved, and early communication between HTA agencies or key stakeholders of drug companies and government regulators was often found insufficient. Regulatory review standards might easily lag behind the technical needs of evaluating clinical trials. For the yield of reliable and high-quality real-world data, appropriate regulatory frameworks and platforms were still developing for data collection and analysis (19, 20).

#### 3.1.2. Set priorities and goals

In recent years, FDA had always been committed to develop, evaluate, and manufacture novel medical products or technologies through strategic planning of advancing regulatory science (8, 21). The EMA and PMDA were equally committed to advancing RS to create a regulatory environment for pharmaceutical products that supports innovation and the development of solutions to meet human needs (22, 23). China had carried out a comprehensive reform of the drug regulatory system and launched the regulatory science action plan (RSAP) to achieve more scientific and effective drug regulation (24).

Due to the differences in the national circumstances and the history of drug regulation reform, the definitions, visions, and priorities of RS set by different DRAs also differed as shown in Table 1. Regarding RS definitions, the FDA and the PMDA noted the same acceptance of RS and highlighted that RS was a scientific discipline with the goal to provide new tools and technology to speed up the approval of medical products, while the EMA emphasized that RS was a scientific discipline covering the regulatory decision-making throughout the medicine lifecycle. The visions of the FDA, EMA, and PMDA were more global in scope, focusing on the advancement of all humankind and international regulatory development. On the other hand, NMPA's vision was more concerned with meeting the demands of recent advances in regulatory policies, focusing on the reform and innovation of the drug review and approval system, and closely following the frontier of international regulatory innovation.

**Table 1:** Definitions, visions, and priorities of RS in selected DRAs.

	**FDA**	**EMA**	**PMDA**	**NMPA**
Definitions	Science of developing new tools, standards, and approaches to assess the safety, efficacy, quality, and performance of all FDA-regulated products (8)	Range of scientific disciplines that are applied to the quality, safety, and efficacy assessment of medicinal products and that inform regulatory decision-making throughout the lifecycle of a medicine. It encompasses basic and applied medicinal science and social sciences, and contributes to the development of regulatory standards and tools (22)	The science aimed at the optimal introduction into society of new products of science, such as discovered substances and new scientific tools and technologies as well as knowledge (23)	NA
Visions	Advance regulatory science to speed innovation, improve regulatory decision-making, and get safe and effective products to people in need; Driving force as FDA works with diverse partners to protect and promote the health of our nation and the global community (8)	Protect human health, catalyze and enable regulatory science and innovation to be translated into patient access to medicines in evolving healthcare systems (22)	Vision 1: to contribute to the world through regulatory innovation Vision 2: to maximize the common health benefits to other counties/ regions. Vision 3: to share the wisdom with other countries/regions. (23)	1. Based on the actual situation of drug regulatory in China, focus on the reform and innovation of drug review and approval system, and closely track the frontiers of international regulatory development 2. Through a series of innovations including develop a batch of regulatory policies, review technical guidelines, inspections, testing and evaluation techniques, and technical standards after 3–5 years of efforts 3. Effectively address the outstanding issues that affect and restrict innovation, quality, and efficiency of drugs and accelerate the modernization of drug management systems control capabilities (24)
Priorities	1. Modernize Toxicology to enhance product safety 2. Stimulate innovation in clinical evaluation and personalized medicine to improve product development and patient outcomes 3. Support new approaches to improve product manufacturing and quality 4. Ensure FDA readiness to evaluate innovative emerging technologies 5. Harness diverse data through information sciences to improve health outcomes;6. Implement a new prevention-focused food safety system to protect public health 7.Facilitate development of medical countermeasures to protect against threats to U.S. and global health and security 8. Strengthen social and behavioral science to help consumers and professionals make informed decisions about regulated products (8) Strengthening the global product safety net (25)	1. Catalyzing the integration of science and technology in medicines development 2. Driving collaborative evidence generation- improving the scientific quality of evaluations 3. Advancing patient-centered access to medicines in partnership with healthcare systems, 4. Addressing emerging health threats and availability/therapeutic challenges 5. Enabling and leveraging research and innovation in RS (22)	1. Taking the lead, and disseminating the information around the globe 2. Promotion of international regulatory harmonization and global cooperation 3. Increase efficiency of inspections for international work-sharing 4. Contribution to international regulatory harmonization activities 5. Provision of information and training programs that are essential for building regulatory capacity in partner countries (23)	First batch of 9 projects 1. Research on technical evaluation and supervision system of cell and gene therapy products 2. Research on safety evaluation and quality control of nanotechnology drugs 3. Research on safety evaluation of clinical practice-oriented traditional Chinese medicine 4. Research on safety monitoring and evaluation methods of post-marketing drugs 5. Research on technical evaluation of drug-device combination products 6. Research on safety and efficacy evaluation of artificial intelligence medical devices 7. Regulatory science research on new medical device materials 8. Methodological research on the application of real-world data for clinical evaluation of medical devices 9. Research on the evaluation method of cosmetics safety (24) Second batch of 10 projects 1. Research on the effectiveness and safety evaluation of Chinese medicine and quality control of the whole process 2. Research on evaluation systems and methods for stem cell and gene therapy products 3. Research on evaluation methods of real-world data-supported Chinese medicine, rare disease therapeutic drugs, innovative and clinically urgent medical devices 4. Research on the evaluation of diagnostic and therapeutic products for new and emergent infectious diseases 5. Research on the evaluation of safety, efficacy, and quality control of nano-class innovative drugs and medical devices 6. Research on the evaluation of innovative medical devices based on remote transmission, flexible electronic technology, and medical robots 7. Research on the evaluation of the safety and efficacy of new biomaterials 8. Research on technical guidelines for new raw materials for cosmetics and research on cosmetic safety monitoring and analysis of early warning methods 9. Research on new tools, new standards, and new methods for the evaluation of common diseases and diseases of malignant tumors 10. Research on drug and medical device alerting techniques and methods (26)

As for the priorities, each of these four DRAs had decided their own priority areas for RS development and thus set specific objectives which might be technology-based (e.g., toxicology and clinical evaluation), process-based (e.g., partnership with healthcare systems and high-quality consultation services) or product-based (e.g., drug–device combination products). The FDA emphasized the adoption of RS to improve the assessment of the safety of medical products and promote product innovation. The convergence of regulation and innovation was the major focus of the EMA. The PMDA highlighted the need to improve the efficiency of global convergence and regulatory activities. As for NMPA, it is committed to effectively addressing the outstanding issues that affect and restrict the innovation, quality, and efficiency of drugs.

### 3.2. Step 3: behavioral and environmental diagnosis

The development path of medical products (including drugs, biological products, and medical devices) had been increasingly challenging, inefficient, and costly. Innovators in this context tended to focus on medical products with high potential market returns; however, developing products that address critical public health needs, rare diseases, and personalized treatments was increasingly challenging. Without enough new tools and concepts created with applied science, developers or scientists could only use old scientific tools and ideas to evaluate candidate medical products. After investing a lot of time and resources, most of the R&D products that entered clinical trials failed. For successful product candidates, the cumbersome review approach and process made the road to market lengthy, costly, and inefficient. This was particularly evident in the reports of Critical Path Initiatives published by the FDA. As reported, the number of new drug and biologics applications submitted by companies to the FDA fell sharply, and the number of applications for innovative medical devices also decreased, but the costs of pharmaceutical product development had soared rapidly since the beginning of this century (21). Although similar reports published by other DRAs, such as the EMA, PMDA, or NMPA, were not identified, regulatory challenges were common across those agencies driving the development of the adoption of RS (27–29).

### 3.3. Step 4: educational and ecological diagnosis

Regulators' perceptions and attitudes toward the current medical product development process and the existing deficiencies of scientific tools and ideas from regulatory authorities constituted predisposing factors. DRAs reinforced and enabled behavioral changes by expanding collaboration with the larger scientific community and issued related actions, plans, and policies. The FDA was found to be the only DRA that had a traceable, well-reported track record of how RS was developed, adopted, and advanced. As such, the FDA's experiences were used primarily to inform this part of the result. In this study, we traced back to the FDA's Critical Path Action Report and Checklist Report to explore the education and ecological diagnostics that led to RS (21, 30–32).

#### 3.3.1. Predisposing factors

Advancing biomedical technologies had brought hope to prevent, treat, and even cure more diseases. However, regulators and stakeholders were increasingly concerned that new basic science discoveries might not quickly yield more safety, effective, and affordable pharmaceutical products for patients. At the same time, with the costs and difficulties of medical product innovation continuing to grow, there was a concern that the development of advanced therapies would be stagnated and declined.

Advanced product development science was needed by drug regulators to address these concerns. The lag in the process of medical product development urgently required a new product development toolkit that incorporated a robust scientific and technological approach to improve predictability and efficiency along the Critical Path, such as computer-based predictive models and new clinical review techniques. A knowledge base was also needed to be built to grasp ideas from biomedical research and reliable insights into the pathway in patients.

#### 3.3.2. Reinforcing factors

Working together to address the above concerns and challenges has received a positive response from many accomplished scientists from academia, government, and industry. A coordinated effort by the stakeholders is needed to apply the new biomedical science to medical product development in order to promote the modernizing of the Critical Path. When serious issues arise during development or common problems persisted, FDA was devoted to seek collaboration from a wider range of internal and external scientists and refocused its efforts, in order to enhance support for critical programs and ensure that the most important issues were addressed.

#### 3.3.3. Enabling factors

FDA's standard-setting process based on the best science facilitated the efficient development of safe and effective new medical therapies. In 2004, FDA planned a Critical Path Initiative to identify and prioritize the most pressing development issues and areas that offer the greatest opportunities for rapid development and public health benefits in the Critical Path White Paper. FDA engaged all stakeholders in the Critical Path work and created a list of Critical Path opportunities to focus its internal efforts to ensure that the most important problems and critical programs are addressed. *The Critical Path Initiative—Report on Key Achievements* in 2009 illustrated the diversity and complexity of Critical Path work and emphasized the public health outcomes (32). This series of initiatives and policies enabled the development of RS.

### 3.4. Step 5: administrative and policy assessment

The RS in FDA kept pace with advanced technologies. The FDA protected consumers by applying the best science to regulatory activities, and each of its medical product centers had taken steps to begin implementing regulatory processes, policies, and internal organizational improvements to address and coordinate regulatory challenges for complex innovative products. In addition, the Office of Regulatory Science and Innovation (ORSI) provided excellence and innovation in strategic leadership, collaboration, coordination, and infrastructure development, ensuring that the FDA continued to have a strong regulatory science foundation (33–36). In advance of its mission to protect and promote public health, FDA began a full institutional reorganization in early 2019 to meet the challenges of rapid innovation in the industry. The FDA's restructuring aligned with the agency's multiple entities to promote regulatory strategic priorities and the specific frameworks of RS in different medical product centers.

**Figure 3:**
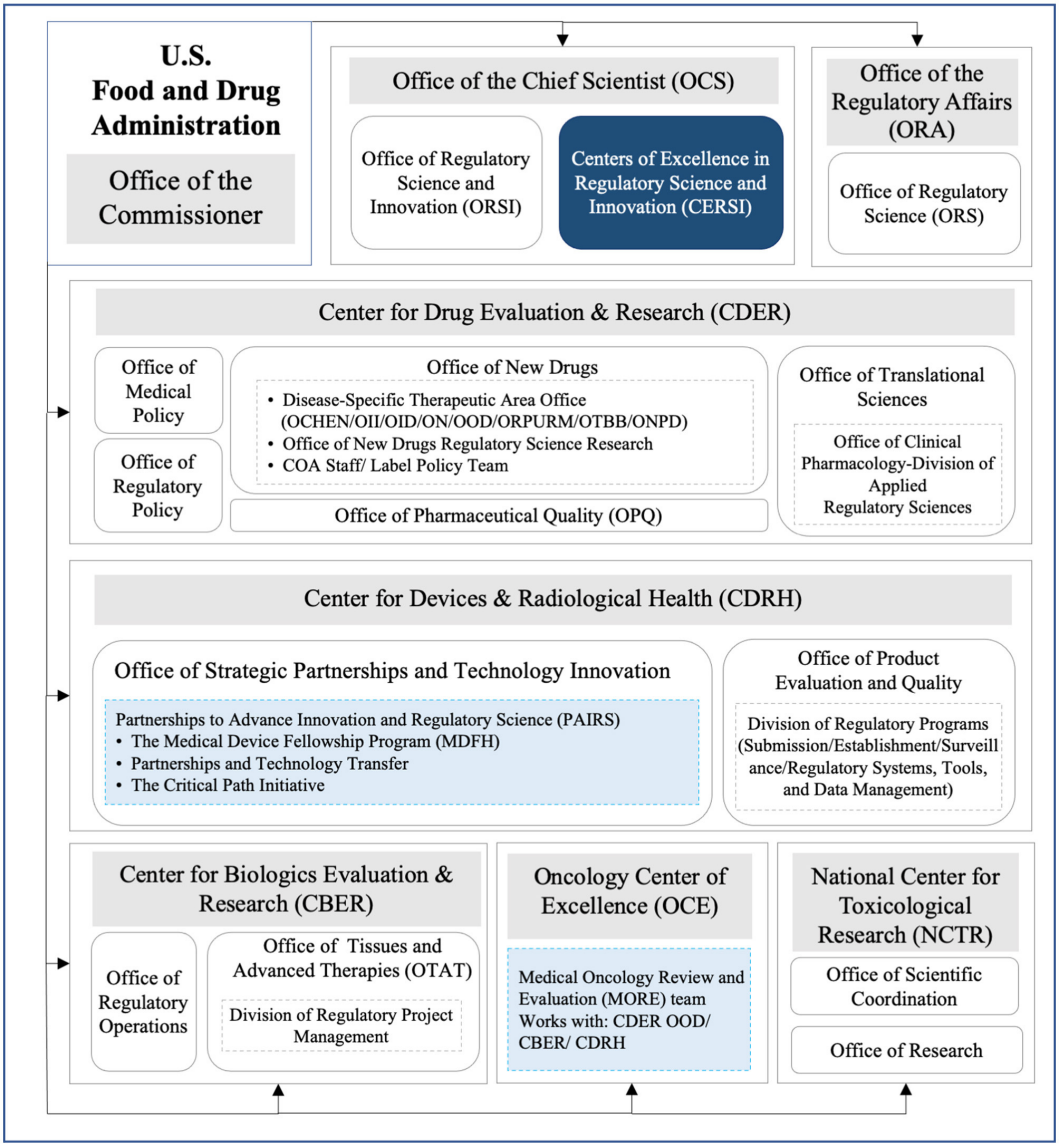
Organization frameworks of RS in different FDA medical product centers. OCHEN, Office of Cardiology, Hematology, Endocrinology and Nephrology; OII, Office of Immunology and Inflammation; OID, Office of Infectious Diseases; ON, Office of Neuroscience; ONPD, Office of Nonprescription Drugs; OOD, Office of Oncologic Diseases; ORPURM, Office of Rare Diseases, Pediatrics, Urologic and Reproductive Medicine; OTBB, Office of Therapeutic Biologics and Biosimilars; COA, Clinical Outcome Assessments; Navy blue box, external cooperation; Pale blue box, internal cooperation.

To name an example, as shown in Figure 3, FDA set up offices of regulatory science, regulatory policy, or regulatory operations in different centers of medical products to facilitate the development of RS internationally. In addition, FDA also established collaborations externally to advance RS. For example, the Centers of Excellence in Regulatory Science and Innovation (CERSI) represented the collaboration between the DRA and external academic institutions to promote cutting-edge scientific research (37) that addressed the needs in drug regulation, and the Partnerships to Advance Innovation and Regulatory Science (PAIRS) were set to acquire the expertise and material resources from different sectors to support scientific and clinical assessments conducted in the offices of CDRH to better promote public health (38). As demonstrated by the actions taken by the FDA, a logical and scientific regulatory system was kept in place to strategically embrace external scientific input and foster internal organizational synergy.

EMA had established special committees for each relevant area to support scientific decision-making in related areas, such as the Committee for Medicinal Products for Human Use (CHMP), the Pharmacovigilance Risk Assessment Committee (PRAC), the Committee for Medicinal Products for Veterinary Use (CVMP), the Committee for Orphan Medicinal Products (COMP), the Committee on Herbal Medicinal Products (HMPC), the Committee for Advanced Therapies (CAT), and the Pediatric Committee (PDCO) (39).

On 14 May 2012, Japan established the Scientific Board as a high-level advisory body to discuss the scientific nature of drug and medical device review (40). The board strengthened the cooperation and exchanges between PMDA and scientists from universities and research institutions and helped PMDA to incorporate the latest scientific knowledge into its services, thereby improving PMDA's review and safety measures and promoting the construction of RS. On 1 April 2018, PMDA established the Center for Regulatory Science to work on solving and simplifying scientific issues, improving audit quality and safety measures, and initiating discussions with each stakeholder by providing information on RS (41). Through the efforts of the Center, PMDA furthered the development of product and post-market safety measures, while actively participating in global regulatory affairs to enable the development of rational medicine in the future (42).

In 2013, the Department of Science, Technology, and Standards of the former State Food and Drug Administration (SFDA) held the first research project initiation meeting on drug regulatory science in Beijing, and the China Society for Drug Regulatory (CSDR) was formally established, which played an active role in promoting the scientific development of drug regulation in China (43). In 2015, the State Council issued the “*Opinions on Reforming the Review and Approval System for Drugs and Medical Devices*”, and the former SFDA decided to use Peking University as a platform to apply for the establishment of the Asia-Pacific Economic Cooperation (APEC) Regulatory Science Center of Excellence (CoA) (44).

### 3.5. Step 6: implementation

**Figure 4:**
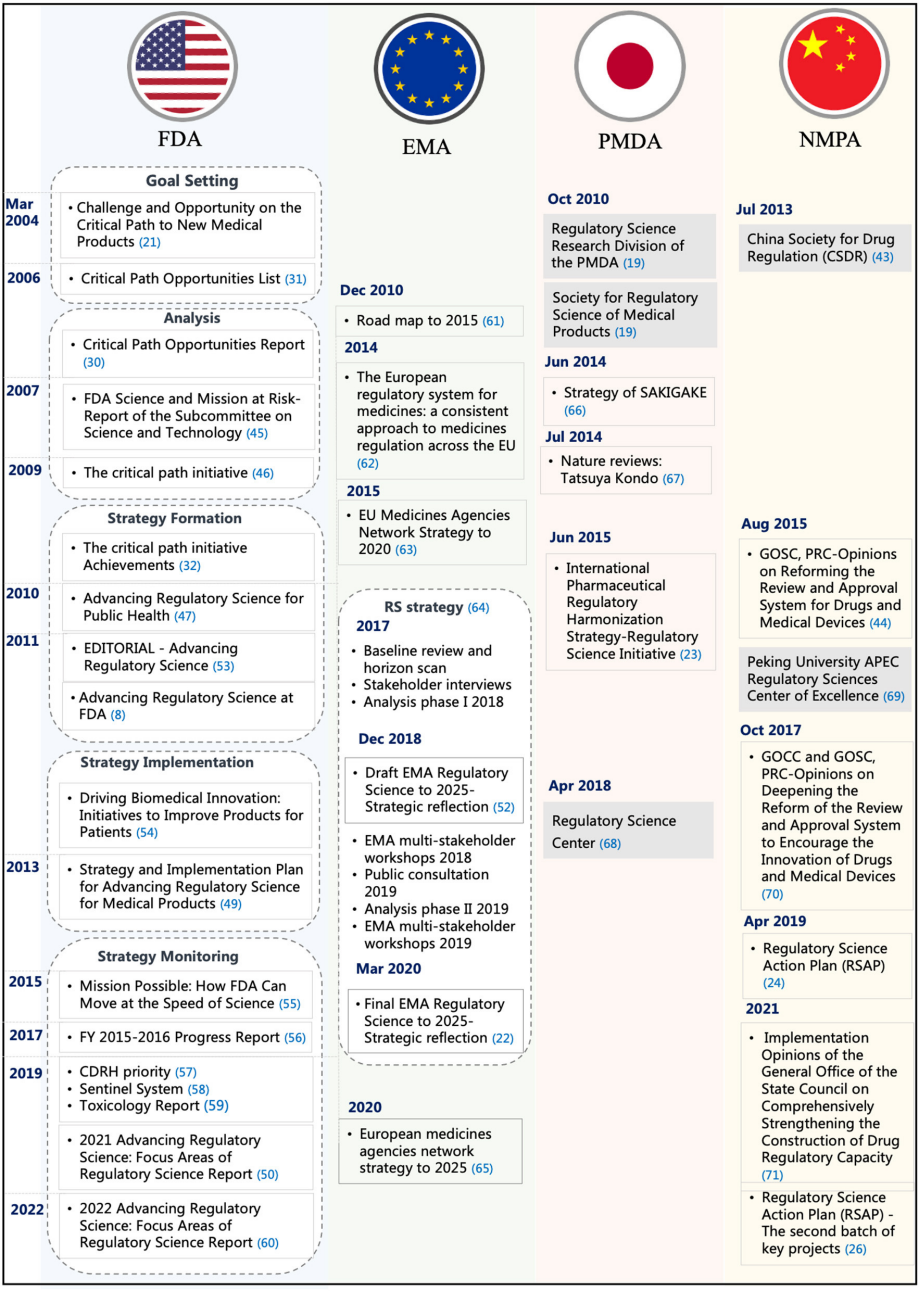
The implementation process of RS in the different countries.

This step explored the implementation of RS in the selected DRAs. Figure 4 depicts the different implementation processes of RS in the FDA, EU, Japan, and China. Since 2004, the FDA had been issuing Critical Path reports and launching Critical Path Initiatives to help accelerate medical product development and review. The regulatory challenges reported in the Critical Path Initiative gradually became a major driver of innovation for the FDA. FDA issued a series of reports that analyze the scientific progress, challenges, and achievements from 2007 to 2009 (45, 46). In 2010, the report Advancing Regulatory Science for Public Health first proposed the basic structure of RS. Since then, FDA's regulatory science shifted from the analysis stage to the strategy formation stage (47). In August of the same year, the FDA released the Advancing Regulatory Science at FDA Strategic Plan, which proposed eight priority areas to advance regulatory science and thoroughly modernize science and technology used in the development and evaluation of pharmaceutical products and planned to transform the regulatory concept of products such as the development, evaluation, manufacturing, and application of medical products by building the Critical Path Model (48). In 2013, the FDA further released the Strategy and Implementation Plan for Advancing Regulatory Science for Medical Products white paper and added a ninth priority area (49). In 2020, recognizing that the science and technology underpinning FDA-regulated products evolved significantly since 2011, FDA formed an agency-wide committee to develop an efficient way to communicate its RS activities. The committee developed the report Advancing Regulatory Science at FDA: Focus Areas of Regulatory Science (FARS) to identify and communicate areas FDA has identified as needing continued targeted investment in RS research to fulfill FDA's regulatory and public health mission (50, 51).

EMA and PMDA also published their strategic plans for RS. On 19 December 2018, EMA first published the *EMA Regulatory Science Strategic Plan 2025* (Draft for Comment), which defined RS and informed regulatory decision-making throughout the lifecycle of medicine (52). *The strategic plan* covered five strategic goals and provided core recommendations and actions based on the goals. The overall top five core recommendations were fostering innovation in clinical trials, promoting the use of high-quality real-world data (RWD) in decision-making, reinforcing patients' relevance in evidence generation, contributing to HTA's preparedness and downstream decision-making for innovative medicines, and supporting developments in precision medicine, biomarkers, and “omics.” After continued consultation and multi-stakeholder participation, in March 2020, the EMA Management Committee officially released the “*EMA 2025 Regulatory Science Strategic Plan*” (22).

In June 2015, PMDA released the *International Pharmaceutical Regulatory Harmonization Strategy—Regulatory Science Initiative* (hereinafter referred to as the “*Initiative*”). The *initiative* mentioned that RS is the foundation of PMDA's activities (23). It was also in this year that China's State Council issued the “*Opinions on Reforming the Review and Approval System for Drugs and Medical Devices*,” which marked the launch of the comprehensive authorization reform plan. Since then, the Chinese government has successively issued hundreds of policies, initiatives, and announcements in order to achieve a more scientific and effective drug regulatory system. The NMPA officially released the first batch of the regulatory science action plan (RSAP) in 2019 and the second batch of RSAP key projects in 2021 (24, 26).

### 3.6. Step 7: process evaluation

In the process of advancing RS, all the selected DRAs focused on staff training and education, as well as internal and external communication and cooperation. The full description could be seen in Table 2. The successful advancement of RS in the FDA focused on its strong regulatory science culture and infrastructure, with an emphasis on government agency partnerships, staff training and professional development, direct funding mechanisms, and public–private partnerships (PPP). The EU supported member state regulators through the establishment of Innovation Networks and created a joint horizon scanning system to provide policymakers, healthcare providers, and patients with access to emerging medical technologies that have a significant impact. At the same time, the EU actively promoted Good Regulatory Practices (GRP) to facilitate coordination and cooperation among multiple forces. PMDA placed great emphasis on streamlining approval time, funding support, and big data utilization. Through the implementation of the “SAKIGAKE” strategy, the “Sakigake designation system and “Senkuteki Iyakuhin” (legislation of system) were typical examples of PMDA accelerating the evaluation of innovative drugs (72, 73). NMPA focused on expanding the education and training and building the RS talent pool and had since cooperated with universities and research institutes to establish a number of RS research centers to promote talent training and scientific research for regulatory capacity building.

**Table 2:** Process evaluation of RS in selected DRAs.

**DRAs**	**Specific process evaluation**
FDA	**Training and education** 1. In-house science base Internal collaborations of scientific and professional meetings and conferences • Academic institutions invited to provide courses, workshops, and seminars 2. Network of collaborations • FDA staff participating in academic activities and initiatives • Commissioner's Fellowship Program brings talented young scientists from academia, industry, and government to FDA to learn about RS
	**Communication and cooperation** 1. Academic, for-profit, non-profit, or government partners, international scientific • NIH-FDA: FDA cooperated with NIH to lead RS Requests for Applications to support the development of RS disciplines. 2. Regional academic collaboration with a Center for Excellence in Regulatory Science and Innovation (CERSIs) to support RS research, training, scientific exchanges, and professional development • University of Maryland: the cultivation of RS talents and research on several topics • UCSF-Stanford: working together to improve the development and approval of effective medical products • Johns Hopkins University, Yale-Mayo Clinic: create an infrastructure to support and strengthen several areas of the FDA's Regulatory Science Strategic Plan • Yale-Mayo Clinic: regularly collect real-world data combined with other databases from other clinical trials to inform regulation
	**Direct funding mechanisms** • Office of Critical Path Programs within the OC direct support scientific programs: grants, cooperative agreements, and contracts • Developing new bioequivalence methods • Fostering innovation approaches to toxicology and biomarker development • Awarded cooperation agreements to institution to establish CERSI to develop master's program
	**Public-private partnerships**: Partners with patient advocacy groups, professional societies, charitable foundations, industry members, trade organizations, and academic institutions. • Patient Reported Outcome (PRO) Consortium • Critical Path to TB Drug Regimens (CPTR) Consortium • Medical Device Innovation Consortium • The Reagan-Udall Foundation (RUF)
	**Internal RS research activities:** Competitive Intramural Funding Programs, Laboratory-based research, Research based on analysis of regulatory data • Medical countermeasures initiative funding programs • Advanced portable screening technologies for drug ingredients • FDA works with sponsors and manufacturers of artificial pancreas devices to access information from regulatory files
EMA	**Training and education** In-house staff professional training and international training programs • Fellowship programs involve exchanging staff for a short period of time to enable the EMA and non-EU regulators to exchange best practices, enhance mutual understanding, and work together more closely
	**Communication and cooperation** 1. Academic cooperation • EMA-MIT: EMA has launched a collaborative project with MIT on RS to enable the “staggered” and “progressive” approach to drug approval and to incorporate patient assessments of health outcomes and risk appetite for benefits into regulatory decisions. 2. International regulatory Cooperation • Activities with FDA, Japan, Canada, Australia, Switzerland, and WHO part of EMA daily work •~80% of all products going through EMA committees have some discussion at international level • 8–10 international calls per week • host 3–4 international visitors per month • New countries and regions emerging as important players, especially China, India, Brazil, and Africa • Bilateral and Multilateral
	3. HMA-EMA EU Innovation Network (established in 2016) • 24 agencies develop/establish innovation offices as a network • Share knowledge, best practices, and seamless support to innovators at local and EU level
	**Joint horizon scanning system** • Identify, filter, and prioritize new and emerging health technologies with a considerable predicted impact on health, costs, society, and the healthcare system
	**Promote WHO Good Regulatory practices (GRPs)** • take into account assessments done by others • retain responsibility for your own decisions • EU-Canada Comprehensive Economic and Trade Agreement (CETA) with Canada and the EU-US Transatlantic Trade and Investment Partnership (TTIP) with the United States
PMDA	**Internal and external training projects** • Establish the “Asian Training Center for Pharmaceuticals and Medical Devices Regulatory Affairs” to provide training • Dispatch PMDA staff members to partner regulatory authorities and conduct on-the-job training • Conduct training on guidelines agreed at ICH, IMDRF, IGDRP, ICCR, PIC/S, etc., • Knowledge and information needed in Asian countries and BRICs
	**Communication and cooperation** 1. Academic cooperation • Comprehensive partnerships agreements Partnerships with medical schools and national advanced medical centers, personnel exchange, and cooperative activities 2. Strengthening the communication with overseas regulatory authorities • Expand the collaboration between regulatory authorities in Japan and the U.S., the EU, and other countries under the Confidential Arrangements and expert area clusters • For medical devices, continue information exchange with the U.S. FDA, by way of the Harmonization By Doing (HBD) activities • Develop robust evidence in cooperation with foreign regulatory authorities, especially for orphan designated products • Continue personnel exchange program with foreign regulatory 3. Streamline international collaboration in GXP/QMS inspections • GMP inspections: Contribute to preparation of the Pharmaceutical Inspection Cooperation Scheme (PIC/S) guidelines and conduct of training programs, and promote collaboration with foreign regulators as a member of the PIC/S • QMS inspections: Become the formal member of MDSAP Pilot, contributing to streamlining of the process of QMS inspections • GCP inspections: Establish a communication channel that allows for open discussion between the US, EU, and Japan 4. Interact with Asian and other countries to enhance mutual understanding and cooperation • Deepen mutual understanding and trust of key ASEAN countries, China/Korea, BRICs, and other countries through bilateral meetings and symposia • Contribute to the improvement of safety measures in the Asian region, by providing Japan's safety information and responding to the diverse needs of partner countries • Collaborate in the areas of consultations and reviews to promote smooth product development in the Asian region • Enhance international regulatory harmonization and cooperation for over-the-counter (OTC) drugs by proactively participating in Self-CARER
	**Streamlining approval time** • “SAKIGAKE” Strategy to lead the world in the practical application of innovative medical products (2014) • “Senkuteki Iyakuhin (Pioneering drugs)”- legislation of “Sakigake designation system” (2019)
	**Funding for RS** • Japan Agency for Medical Research and Development • Awarded projects including quality test, non-clinical study, clinical trial, approval review, and post-marketing
	**BIG DATA utilization** • MID-NET Project (network of 10 base hospital databases of 23 hospitals): analyze electronic health records, insurance claims data, diagnostic procedure combination data, and experimental test results.
NMPA	**Training and education** 1. Training regulatory talents through RS research bases and key laboratories • Well-known universities and top scientific research institutions • Systematically carry out basic theoretical research on drug RS • Promote the construction of RS disciplines • Cultivate leading talents in RS 2. International training • Committee for International Pharmaceutical Regulatory Science Education Promotion (CIPRSEP): the first seven members signed the agreement, including China Pharmaceutical University, Fudan University, Peking University, Shenyang Pharmaceutical University, European Association of Pharmaceutical Regulatory Science (TOPRO), University of Macau, and University of Southern California, USA
	**Academic cooperation** • Binhai New Area Food and Drug Administration- Tianjin Institute of Pharmaceutical Research: Tianjin Binhai Food and Drug Regulatory Scientific Research Center • NMPA-Sichuan University: Medical Device Regulatory Science Research Center • NMPA-China Academy of Chinese Medical Sciences (CACMS): research center for regulatory science of Chinese medicine • NMPA- Beijing University of Chinese Medicine (BUCM): Institute of Regulatory Science of Chinese Medicine • NMPA CDE-Tsinghua University: Institute of Drug Review Science and Regulatory Science • APEC Regulation Harmonization Steering Committee-Peking University and Sichuan University: Centers of Excellence • NMPA-Shenyang Pharmaceutical University (SYPHU): a strategic cooperation framework and SYPHU Institute of Regulatory Science

### 3.7. Step 8: impact evaluation

Through the continuous efforts of regulatory bodies, the application of RS had a great impact on different procedures. Currently, advances in the adoption of FDA medical product RS included integrating new science into the regulatory process, more professional training, and infrastructure building to enhance the evaluation of RS and advocate the adoption of new science and technology. For the EU EMA, it increased the interaction with the pharmaceutical industry throughout the drug development lifecycle by supporting patient involvement throughout the drug research and development process. By incorporating RS, the EMA also translated scientific regulation into better processes through a process for writing guidelines. PMDA proactively created guidelines leading to international regulatory harmonization and established different institutional departments in specialized fields.

RS in China started later than previous DRAs, but through two sets of RSAP, RS has gradually become a mature discipline in China. Since 2019, NMPA had issued a series of new systems, tools, standards, and methods for drug review and supervision to promote the innovation of drug regulation systems and improve the regulatory capacity. NMPA relied on universities to establish various special research projects, focusing on the key and difficult issues of the regulation of drugs, medical devices, and cosmetics. NMPA actively held and participated in domestic and international regulatory activities to build a platform for common exchanges and discussions among regulatory authorities, industry, and academia. Table 3 describes the detailed information.

**Table 3:** Impact evaluation of RS in selected DRAs.

**DRAs**	**Specific impact procedures**
FDA	**Integrating New Science into the Regulatory Process**1. Evaluating and Adapting New Science for Regulatory Purposes a. Advisory Committee general matter meetings: Cell Lines Derived from Human Tumors for Vaccine Manufacture b. Formal processes that target the evaluation of new science: voluntary exploratory data submissions c. Workshops: CERSI workshops d. Working groups: industry RS work group meeting 2. Applying New Science to the Regulatory Process a. Development and updating of guidance and regulations: accelerated approval program b. Actions prompted by new science: labeling changes; Medsun c. Product-specific advisory committee meetings: in-home HIV test d. Formal processes for regulatory acceptance of emerging scientific developments: drug development tool qualification process e. Consultations and collaborations with international bodies: establish international standards for biologics(PAHO-WHO)
	Training and Professional Development 1. Providing internal staff training and education programs 2. Offering educational programs for FDA staff outside the agency 3. Sponsoring lectures and seminars featuring global scientific thought leaders 4. Building cross-Agency collaborations 5. Expanding extramural collaborations 6. Providing training and education for external organizations
	**Building Infrastructure** to Enhance the Evaluation of Regulatory Submissions and Support the Adoption of Emerging Science and Technology 1. Developing and/or integrating new data standards, and computer hardware and software tools for data receipt, analysis, evaluation, and visualization, to facilitate efficient, effective, and consistent review of complex data *(FDA's Technology Modernization Action Plan, TMAP)* a. The Janus Clinical Trials Repository (CTR) – data warehouse application that supports automated extraction, transformation, loading, management, and reviewer access to standard clinical trial data. b. The DataFit program – leverage standard data to advance the review progress c. The Nonclinical Information Management System (NIMS) – a software tool for non-clinical study data d. The High-performance Integrated Virtual Environment (HIVE) - cloud-based environment for storage and analysis of sequencing data generated using high-throughput technologies e. Medwatch Plus System - online adverse event and reporting system 2. Science and Research Infrastructure – Investments in Key Technologies to Prepare for Regulatory Evaluation of Innovative Medical Products and Enhance Evaluation of Existing Licensed Products *(FDA's Technology Modernization Action Plan, TMAP)* a. Critical core technologies: high-throughput sequencing, high-resolution nuclear magnetic resonance and mass spectrometry, multi-color flow cytometry, and ultrahigh resolution confocal microscopy b. Nanotechnology Core Facilities: two core nanotechnology facilities managed by NCTR ORA and CDRH c. Computational Modeling: FDA engineers have established a high-performance computer facility to develop models for emerging device technologies.
EMA	**Patient involvement in regulatory processes** 1. Innovation Medicines Initiative (IMI): EUPATI collaboration with 33 organizations from patient organizations, universities, non-profit organizations, and pharmaceutical companies. 2. Guidance for Patient Involvement in HTA: support the integration of patient involvement throughout the entire drug research and development process and contribute to the interaction with the pharmaceutical industry throughout the drug development lifecycle related to the human use of medicines
	**Translating regulatory science into better processes** 1. Integrate RS through guideline, Public statement, Reflection paper, Question & Answer, Addendum to guideline, and Recommendations/procedural advice 2. Procedure for writing a guideline a. Public consultation: usually 2-3 months b. Implementation: 6 months post-publication c. Publication of a CP to publication of final guideline:2-3 years d. Training for assessors/experts, workshops e. Revision – to be considered on annual basis f. Communication – under “what's new” section of EMA website g. set up Guideline Consistency Group
PMDA	**Establishment of institutional departments in specialized fields**•Division of Drugs: nanomedicines and DDS •Divisions of Biological Chemistry & Biologicals and Organic Chemistry: specialty peptides •Division of Molecular Target & Gene Therapy: oligo-nucleotide drugs/ gene therapy drugs •Division of Cell-based Therapeutic Products: tissues and cells for cell-based therapy including iPS cells •Divisions of Cell-based Therapeutic Products and Biological Chemistry & Biologicals: detection, inactivation, and removal methods of infectious agents contaminated in raw materials or products •Division of Medical Devices: new biomedical materials including biocompatibility •Divisions of Medicinal Safety Science, Molecular Target & Gene Therapy Products, and Biochemistry: Biomarkers for personalized medicine, molecular diagnostics, and radioactive diagnostic agents •Division of Biological Chemistry & Biologicals: antibody drugs and advanced modified protein drugs•Division of Pharmacology: safety pharmacology tests using iPS cells
	**Proactively propose to create guidelines leading to international regulatory harmonization** 1. ICH: continuously make efforts to propose and draft harmonized guidelines by participating countries 2. IMDRF: lead the establishment of the mid-term strategy for activities up to 2020 and draft harmonized guidelines 3. IGDRP: promote the consistency of regulations set forth for generic drugs in Japan with international regulations 4. OECD/GLP: actively lead the initiative as a chair, to strive to enhance the scope and up-skilling of participating countries 5. ICMRA: promote activities for the formal inauguration of ICMRA, and contribute to up-skilling of the regulators 6. APEC LSIF RHSC: promote regulatory harmonization and the establishment of training programs 7. ISO/IEC: propose new topics to standard developing bodies 8. ICCR: contribute to the harmonization of cosmetics regulations from the technical perspective
NMPA	**New systems, new tools, new standards, and new methods for drug review and regulation** 1. Update the law: Vaccine Administration Law and Drug Administration Law 2. Update the regulations: Regulations on the Supervision and Administration of Medical Devices, Regulations on the Supervision and Administration of Cosmetics, Measures for the Administration of Drug Registration 3. New tools, new standards, and new methods: The first batch of RSAP developed 103 items, of which 45 have been published (As of 2021/10)
	**Rely on research institutions to lay out key research projects on RS** 1. 14 RS bases and 117 RS key laboratories: set up supported by RSAP 2. Chinese medicine: the CACMS, BUCM, and Tsinghua University make use of their own characteristics and advantages of complete disciplines and national R&D platforms to integrate multidisciplinary knowledge systems, use multifaceted technical support to guarantee the promotion of high-quality development of the Chinese Medicine industry 3. Medical device: Sichuan University RS research base focus on innovative products and new technologies in the national medical device development plan, take the scientific research on the regulation of biological materials and implantable devices as an entry point, and gradually expand to establish a regulatory science system covering the entire medical device 4. Clinical trials: NMPA partnered with APEC to establish the Center of Excellence in Regulatory Science in 2015, which focus on capacity building in the fields of multicenter clinical trials and GCP inspections for APEC members
	**Hold and participate in internal and external regulatory activities**•The National Postdoctoral Forum on Drug Safety and Regulation (NMPA)•China Conference of Drug Regulatory Science (China Society for Drug Administration)•China Pharmacovigilance Conference (NMPA)

### 3.8. Step 9: outcome evaluation

As early as 2013, the FDA issued a clear indication in the white paper “*Strategy and Implementation Plan for Advancing Regulatory Science of Pharmaceutical Products”* for measuring outcomes in advancing and adopting RS in the United States, which was also a unique point of the FDA in the development of RS. FDA measured the metrics about the process of advancing RS through internal RS activities and external communications and collaboration. Three main metrics were used to measure the effectiveness of the impact of RS, including evaluating scientific training and professional development, integrating new science into the regulatory process, and building infrastructure to evaluate emerging science and technology. Enhanced staff capacity increased regulatory efficiency and transparency, and improved infrastructure in which translation of science and technology was anticipated as the key primary outcomes of the RS development. Other DRAs had not yet formally documented specific metrics to measure the outcomes of advancing the RS.

The FDA launched the Sentinel Initiative in 2008. In September 2014, it gradually transitioned from a mini-sentinel pilot phase to the full Sentinel System, which was used to monitor the safety of regulated products as a national electronic system and had developed the world's largest multi-site distributed data dedicated to the safety of medical products. In 2019, the FDA released the “Sentinel System: FIVE-YEAR STRATEGY 2019–2023,” which aimed to guide the path of the Sentinel System from 2019 to 2023. The main direction was to strengthen and expand the foundation of the Sentinel System (i.e., data, infrastructure, operations, and technology). The progress had also seen the enhancement of the security analytics capabilities of the Sentinel System by leveraging advanced technologies in data science and signal detection. At the same time, leveraging the Sentinel System to accelerate access and broaden the use of real-world data to generate real-world evidence was also reported. Finally, the stakeholder ecosystem of the Sentinel System was further expanded in pursuit of more effective coordination of national resources (58).

FDA's advances in RS could be implemented in many ways to streamline the device development process. The FDA Center for Devices and Radiological (CDRH) released the report “Regulatory Science in FDA's CDRH: a Vital Framework for Protecting and Promoting Public Health,” which focused on advancing the RS of medical devices and fostering innovation by substantially reducing the cost and time required for medical devices while feeding back accurate information to evaluate their safety, quality, and performance (74).

## 4. Discussion

This review analyzed how RS was developed, adopted, and advanced by the FDA, EMA, PMDA, and NMPA. By comparing their experiences systematically, an implementation science framework specific to RS development was also developed that is pragmatic and comprehensive for reference by other DRAs and research institutes. Evidence about the adoption, advancement, and monitoring of RS by the studied DRAs was presented to rationalize the formation of the model. The DRAs in this study acknowledged the basic principles of the RS discipline in general terms and identified their respective priorities and specific objectives for the development of RS according to their needs and priorities. Regulatory workforce capacity building and internal and external scientific exchange and collaboration were the primary means most commonly employed by DRAs to advance and adopt RS. The practical outcomes of adopting RS in terms of regulatory performance turning into benefits to public health, patient outcomes, and translation of drug research and development should be further defined to allow monitoring and evaluation for continuous improvement in the implementation of RS. Continuous government commitment and key stakeholder engagement and collaboration are essential for the dynamic development of RS to better prepare DRAs for the mounting challenges of drug regulation.

### 4.1. The vital role of the government in advancing RS

In recent years, there has been a meaningful shift in the view of professionals and the government in its use of research evidence for policy decision-making (75). Evidence-informed policymaking has been widely considered in a variety of fields, including the healthcare arena, social science, and business management. The policymakers make use of this approach, which ensures that decision-making is well-informed by the best available research evidence, for systematic and transparent access to evidence as a prerequisite for the process of policymaking (76, 77). Adopting RS to obtain scientific evidence and apply it in the decision-making process has been used by many governments (78–80). It has been found that providing regulators with the resources, knowledge, and evidence they need for the decision-making process helps to improve their identification of the most pressing practical social needs and strengthen their regulatory capacity (81, 82). Practically speaking, the development of technological and industrial developments often puts forward higher challenges and requirements for regulation, thereby promoting the reform of the legal system, including the 2019 coronavirus pandemic, the breakthrough developments of advanced therapy medicinal products, and the digital transformation of the healthcare industry.

### 4.2. Dynamic evaluation of the development of RS using implementation science framework

To be able to deliver drug regulators with expectations for advancing RS, a systems thinking approach guided by implementation science might offer a clear roadmap that helps translate the outcomes of RS into the formulation of new technique guidelines, new tools, or new scientific methods (83). The adoption of PPM is of particular importance in facilitating the translation of concepts into action for the sustainable development of RS (14). Addressing the gaps in regulatory behaviors and environment is part of a highly complex undertaking involving not just the knowledge, attitudes, and skills of regulators or DRAs but also communication and cooperation with other counterparts throughout the whole health system, as well as different resources, services, policies, and plans in the larger global pharmaceutical markets (84). The use of systems thinking approaches encourages relationship-building across various functionalities of the DRAs so as to achieve a common set of relevant goals and objectives on drug regulation (85).

Predisposing, reinforcing, and enabling factors are unique to a different national context that should be clearly and systematically identified for better planning of RS adoption. The best example is the Critical Path Initiative issued by the FDA which served as a precursor to its Regulatory Science Strategic plan. In the 2004 Critical Path Report, the FDA presented its diagnosis of the scientific challenges underlying the medical product problem and aimed to identify the gaps between the development of medical products and clinical use in Critical Path actions.

**Figure 5:**
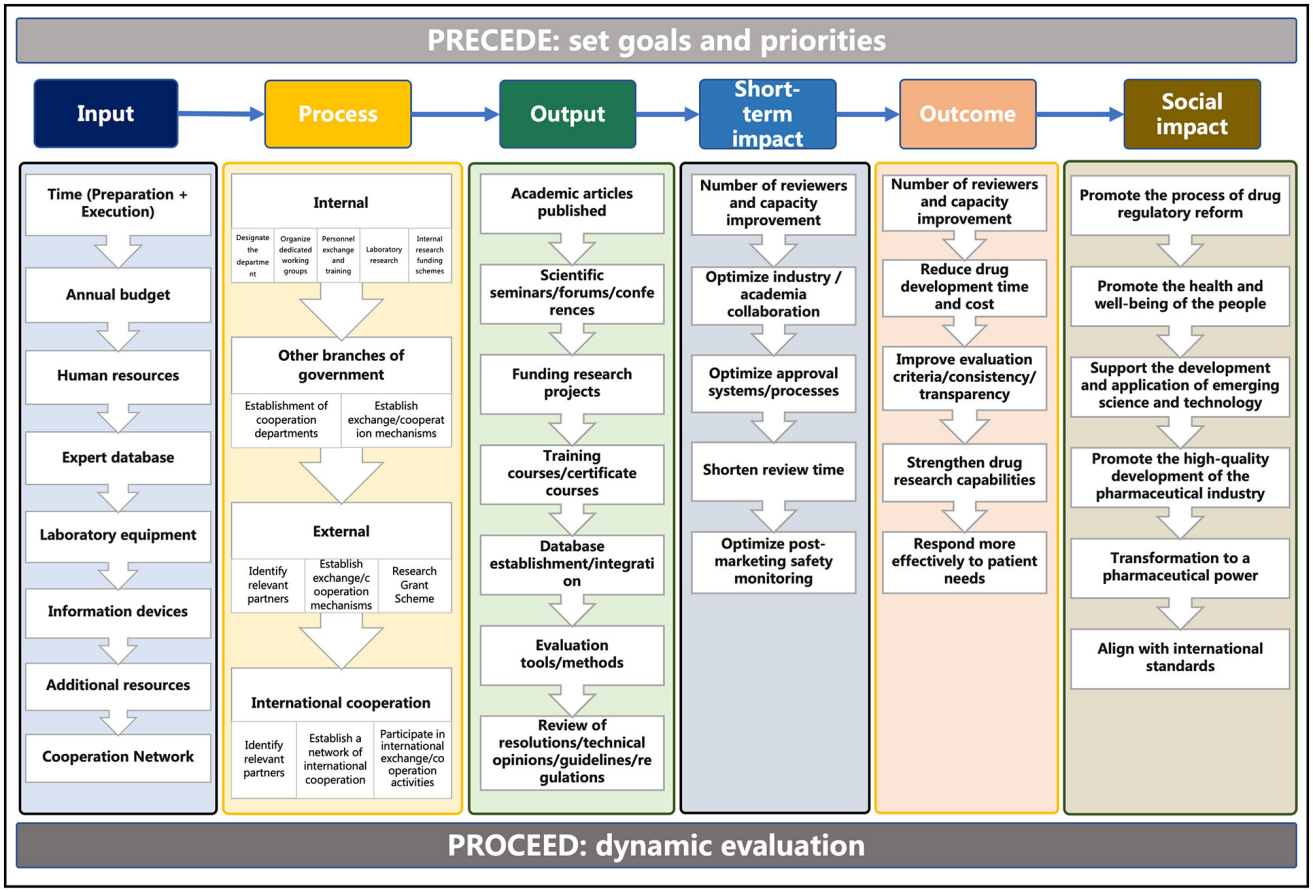
Elements of the development of RS based on the PPM.

As depicted in Figure 5, we summarized the planned RS visions, priorities, and goals of the PRECEDE phase and identified the elements of each step by analyzing the experience of the development and adoption of RS in the US, EU, and Japan through the PROCEED phase. The continuous investment into capacity building of the regulatory agency in terms of hardware, software, laboratory, expertise, as well as collaboration with stakeholders is the key to implementing RS actions. Through the entire process of the implementation science framework, it is not difficult to find that the development of RS is a dynamic process that should be resilient and responsive to new health threats and healthcare needs as well as advancements in technology and innovation.

The elements of the various stages of regulatory science development summarized based on the PPM framework are also applicable to countries or regions outside of those selected for this study. By analyzing the PRECEDE part, regulatory authorities can clearly identify the comprehensive factors that influence the development of RS, set priorities or specific goals, and then implement RS. By using the PROCEED part to systematically monitor and evaluate the input, implementation process, output, impact, and outcomes of RS. Regulatory authorities are more easily able to identify strengths and weaknesses for continuous quality improvement. In addition, by evaluating the development path of RS in different countries or regions through similar elements, regulatory authorities can engage in cross-comparisons and absorb international advanced experiences, thereby driving the reform process of regulation.

### 4.3. Continuous investment in regulatory capacity building

The development of RS should be considered in a context that can be evaluated. In the outcome evaluation (PROCEED step 9), only FDA has published metrics for measuring the advancement and adoption of regulatory science. The regulatory management system that measures change and demonstrates any outcomes related to changes in regulatory capacity is essential to support the sustainability of interventions or services. A scientific approach is needed to identify a range of factors that may facilitate the adoption of recommended actions and changes in regulatory practice. To this end, performance measurement knowledge and strategies must be adopted and incorporated into regulatory management systems to increase the effectiveness of interventions, while collecting the benchmarking data needed to establish evidence-based improvements (86).

Benchmarking tools have been increasingly used by DRAs to internally measure the performance of the regulation system in order to identify areas of improvement to achieve sustainable capacity optimization. In addition, the assessment of regulators against the international best counterparts likewise helps to inform actions to standardize regulatory practices. These would continuously improve the level of regulatory performance and international recognition. For instance, the Health Sciences Authority (HSA) of Singapore and the Ministry of Food and Drug Safety of the Republic of Korea have reached the highest level (Maturity Level 4) achievable for regulatory system evaluation against the WHO's GBT (87, 88), which provide a reference point for regulatory action by Asian drug regulators.

### 4.4. Strengths and limitations

Based on the existing concepts and frameworks for conducting documents and literature retrieval, evaluation, and evidence synthesis, this study shows a systematic view of the process of developing and adopting RS in the FDA, EMA, Japan, and China. The use of an implementation science framework offers some strengths to advance the development of RS. On the one hand, using the evaluation plan through PPM promotes standardization, consistency, and completeness in evaluating the development and adoption of RS, facilitates cross-regional comparisons, and fosters generalizable regulatory knowledge. On the other hand, the framework has great value in building the evidence base for the input and process involved in implementing RS. The evidence can be used to increase the likelihood of output, outcome, and long-term impacts of implementation. The findings of this study could push the elements of developing RS to a wider range of institutions, groups, regions, or countries.

However, there are still some limitations in the study. First, there are many assessment frameworks existing for implementation science, and it is difficult to use one framework to provide a comprehensive evaluation of how things are evolving. The PRECEDE-PROCEED model has the advantage of clearly linking program design and program evaluation metrics to support continuous process improvement. However, the PPM itself has some limitations, including the narrow definition of steps that requires at times more extensive application. In addition, the PPM has traditionally been applied to population-health programs but may be less applicable to some clinical settings. There is no involvement of access to key stakeholders in the PROCEED evaluation phase. Moreover, the bias in the publications that adopt RS in different international authorities persists, which means, no matter what form of developing or advancing RS by governments, the negative evaluation outcomes are rarely or unlikely to be fully reported. At the same time, some of the data that measures regulatory performance is internally confidential and less easily accessible, which might inevitably affect the completeness of the study findings reported in this review.

## 5. Conclusion

Through the pragmatic application of PPM, the development and adoption of RS can be translated into evidence-based decision-making by DRAs. This study provided a detailed analysis and summary of the elements that needed to be taken into account in the process of advancing RS and helped informed some insights for policymakers when considering whether to adopt RS and how to advance RS. In the future, methodological research on how to measure the outcomes of RS is warranted. The use of benchmarking against international best practices may be useful in providing some insights into different aspects of improved regulatory capacity building for DRAs. Continuous government commitment and key stakeholder engagement and collaboration are essential for the dynamic development of RS to better prepare DRAs for the mounting challenges of drug regulation.

## Data availability statement

Publicly available datasets were analyzed in this study.

## Author contributions

JS and CU planned and designed the study. JS, XC, CU, and HH were responsible for data management and analysis. JS, XC, and CU drafted the manuscript. CU and HH critically reviewed and revised the manuscript. All authors approved the final version of the manuscript and agreed to be accountable for all aspects of this article.
